# Rat retinal function attenuation with IOP elevation is impacted by both blood pressure and intracranial pressure

**DOI:** 10.3389/fphys.2025.1566032

**Published:** 2025-06-18

**Authors:** Sera N. Ganearachchi, Anna K. van Koeverden, Christine T. O. Nguyen, Zheng He, Vickie H. Y. Wong, Bang V. Bui, Da Zhao

**Affiliations:** Department of Optometry and Vision Sciences, The University of Melbourne, Parkville, VIC, Australia

**Keywords:** retina, blood pressure, intracranial pressure, perfusion pressures, electroretinography

## Abstract

**Introduction:**

To consider how blood pressure and intracranial pressure modify the way that retinal function responds to intraocular pressure elevation in rats.

**Methods:**

Six groups of adult Long–Evans rats (n = 7–11 eyes/group, total animals 25) were anesthetized and underwent acute pressure modification. Blood pressure (BP) was measured via a femoral artery cannula and elevated by angiotensin II infusion into the femoral vein. Intracranial pressure (ICP) was set to 0 mmHg, 5 mmHg, or 25 mmHg in three separate groups of rats via a cannula in the lateral ventricle. At each ICP (−5 mmHg, 5 mmHg, or 25 mmHg) and BP setting (normal or high), intraocular pressure (IOP) was increased from 10 mmHg to 90 mmHg in 10 mmHg steps. At each IOP level, ganglion retinal function was assessed using the electroretinogram.

**Results:**

Compared with normal blood pressure groups, animals with high blood pressure had significantly smaller baseline ganglion cell-mediated scotopic threshold responses (STR). Animals with high ICP had larger scotopic threshold response (STR) amplitudes than the normal and low ICP groups. Both high BP and high ICP rendered retinal function less susceptible to IOP elevation; however, the effect was greater for high BP.

**Conclusion:**

Retinal function is critically dependent on ocular perfusion pressure; excessive low or high perfusion attenuates function. The ocular perfusion pressure (BP–IOP) relationship largely accounts for the effect of IOP and BP modulation on retinal function but could not account for differences in ganglion cell function between ICP levels.

## 1 Introduction

The risk of retinal ganglion cell (RGC) loss in glaucoma increases with higher levels of intraocular pressure (IOP). However, IOP is not the only pressure that can impact the health of ganglion cell axons at the optic nerve head; both intracranial pressure (ICP) and blood pressure (BP) can influence RGC axon health (Costa et al., 2009; Flammer et al., 2013; He et al., 2011; Zhao et al., 2014). It is well known that BP can modify the effect of IOP elevation, with low BP (or low ocular perfusion pressure, the difference between BP and IOP) increasing glaucoma risk (Hayreh, 1996; Hayreh, 2001; Hayreh et al., 1994; Leske et al., 2008). Indeed, acute changes in blood pressure can have very immediate impacts on how the eye copes with IOP-related stress. He et al. (2012) showed that high BP was highly protective for retinal function against acute IOP elevation, whereas retinal dysfunction due to IOP elevation was exacerbated in the presence of low BP.

In addition to BP, cerebral spinal fluid pressure (CSFp, which is indicative of ICP), exerted by the CSF-filled subarachnoid space, surrounds the retrolaminar optic nerve and may also have a role in supporting nerve health (Berdahl et al., 2008; Mathieu et al., 2017; Ren et al., 2010; Zhang and Hargens, 2014; Zhang et al., 2015). Growing evidence suggests that lower CSF pressure, including reduced production and turnover, can play a role in glaucoma development and progression. Lower CSF pressure can result in an increased pressure difference between intra- and extraocular compartments on either side of the lamina cribrosa (or translaminar pressure difference). A higher translaminar pressure difference can increase stress and strain on connective and support tissues at the optic nerve head, which can ultimately promote axonal injury (Berdahl et al., 2012; Price et al., 2020; Siaudvytyte et al., 2014; Siaudvytyte et al., 2015). A number of studies have investigated interactions between ICP and IOP and how this impacts optic nerve structure (Santos et al., 2023) and retinal function (Zhao et al., 2015; Zhao et al., 2018). Consistent with the idea that a higher optic nerve pressure difference is worse for the eye, Zhao et al. (2015) used a rodent model to show that lower ICP exacerbated the attenuation of retinal function induced by IOP elevation.

While studies have assessed the effect of different BP or ICP levels on the eye’s response to IOP elevation, there has yet to be an attempt to modify both BP and ICP simultaneously. In this experiment, we will study the effects of acute changes in BP, IOP, and ICP on retinal function (electroretinography) using a rodent model. Specifically, through modification of these three pressures, this study considers which of these pressures have the largest influence on retinal function in an acute setting. A better understanding of interactions between these pressures will provide insights into their potential role in the pathogenesis of glaucoma.

## 2 Materials and methods

### 2.1 Experimental animals

Male Long–Evans rats aged 18 months (equivalent to 45–50 years of human age) were used in this study (n = 7–11 per group). The older age animals target previous reports of age-related increased susceptibility to pressure elevation in rats (Zhao et al., 2018) and susceptibility to glaucoma in human eyes. Animals were housed in a 21°C environment in well-ventilated cages (two rats per box), with *ad libitum* access to water and standard laboratory rodent chow (Barastoc, Melbourne, VIC, Aus). To minimize the potential for any light-induced retinal injury, animal housing lighting was maintained below 50 lux on a 12-h light/dark cycle (on at 7 am, off at 7 pm). In addition, the boxes were rotated weekly over different vertical levels of the housing rack to ensure equal light exposure to overhead lighting.

All experiments were conducted under ketamine and xylazine anesthesia (60:5 mg/kg intraperitoneal injection, Troy Laboratory, Glendenning, NSW, Australia). Body temperature was maintained at 37.5°C ± 0.5°C using a thermostatic pad throughout experiments. Topical anesthesia and mydriasis were achieved with Alcaine (0.5% proxymetacaine hydrochloride, Alcon Laboratories, Sydney, NSW, Australia) and Mydriacyl (0.5% tropicamide, Alcon Laboratories) eye drops, respectively.

### 2.2 Intraocular pressure modification

**FIGURE 1:**
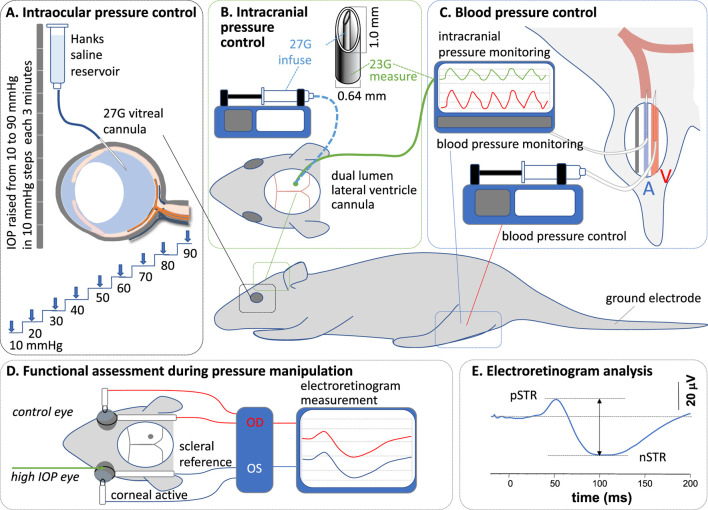
Experimental preparation and protocol. **(A)** At each intracranial and blood pressure combination, intraocular pressure (IOP) was elevated in steps of 10 mmHg, from 10 mmHg to 90 mmHg, via an intravitreal cannula. **(B)** Intracranial pressure (ICP) was measured and controlled via a dual-lumen lateral ventricle cannula. **(C)** Blood pressure (BP) was measured and controlled via a cannula in the femoral artery and vein, respectively. **(D)** Functional assessment was undertaken with electroretinography. **(E)** Response amplitudes were measured between peaks and troughs.

As schematized in Figure 1A, IOP was modified via a vitreal chamber cannula placed as described previously (Zhao et al., 2015). The cannula consisted of a 27G needle connected to a Hanks balanced salt solution (Sigma Aldrich, St Louis, MI, United States) via polyethylene tubing (diameter: 0.8 mm × 0.4 mm, Unomedical, Sydney, NSW, Australia). The reservoir was placed at pre-calibrated heights to control IOP between 10 mmHg and 90 mmHg. A pressure of 10 mmHg was considered the baseline.

### 2.3 Intracranial pressure modification

ICP was manipulated via a custom-made double-lumen cannula placed into the lateral ventricle on the side ipsilateral to the cannulated eye (Figure 1B) (Zhao et al., 2019a). The dual-lumen cannula consisted of a 23G outer (0.6 mm diameter x 19 mm length, Becton Dickinson, Franklin, WI, United States) and a 27G (0.3 mm diameter × 13 mm length, Becton Dickinson) inner needle, which were connected via polyethylene tubing (0.8 mm) to a pressure transducer (Transpac, Abbott Critical Care System, Colorado Springs, CO, United States) for ICP measurement and to a syringe pump (Pump 11 Elite Syringe Pumps, Harvard Apparatus, Holliston, MA, United States) for fluid infusion. The transducer’s signal passed through a bridge amplifier (ML 110, ADInstruments, Colorado Springs, CO, United States) to an amplifier (ML 785, PowerLab/8SP, ADInstruments) for direct and continuous ICP monitoring (LabChart 7, ADInstruments). The syringe pump was set to various flow rates to deliver fluid into the lateral ventricle, maintaining ICP at target levels.

For lateral ventricle cannulation, anesthetized animals were stabilized on a stereotaxic platform (Model 900, David Kopf Instruments, Los Angeles, CA, United States). A 2 cm by 2 cm window of skin was removed to expose the skull. Connective tissue, fat, and muscles around the calvarial area were cleared to allow the coronal sutures to be visualized. A dental burr (0.6 mm diameter) attached to a rotary drill (Model 300, Dremel®, Robert Bosch Tool Corporation, Racine, WI, United States) was used to make a small hole through the skull; 1.5 mm caudal to the bregma and 2 mm lateral to the midline (Paxinos, 2019). The cannula was then inserted perpendicular to the skull, to a depth of 3.5 mm (Zhao et al., 2015), to sit within the lateral ventricle. Corneal hydration was maintained throughout the procedure to minimize cataract formation using Genteal eye gel (Alcon Laboratories). Previous work has shown that the ERG remains stable over time with this approach (Weymouth and Vingrys, 2008).

### 2.4 Blood pressure modification

Blood pressure was monitored using a cannula placed in the femoral artery (Figure 1C) as previously described (Zhao et al., 2018). Continuous monitoring of arterial blood pressure enabled the calculation of mean arterial pressure. A polyethylene cannula primed with heparin (100 units) was inserted 20–30 mm proximally into the left femoral artery, secured with surgical suture (6/0), and connected to a pressure transducer (see above). A second cannula was inserted into the left femoral vein for drug delivery to increase (angiotensin II, 10 mmol/L in saline, AusPEP, Parkville, Australia) blood pressure. Blood pressure was maintained at 90–110 mmHg (without any drug) and above 150 mmHg (with angiotensin II) for normal and high BP groups, respectively, by continuous adjustment of the drug delivery rate using an electronic syringe pump (HA11D, Harvard Apparatus, Holliston, MA, United States). The infusion rate varied between 0.004–0.01 mL/min and was adjusted in 0.002 mL/min increments.

Blood pressure is summarized in Supplementary Figure S1. This shows that baseline blood pressure was similar across groups (one-way ANOVA, F5 = 1.55, p = 0.19) and that blood pressure was stable across the period of ERG recordings for the various groups.

### 2.5 Experimental protocol

Each animal was randomly allocated to two of three ICP levels (low: −5 mmHg, baseline: 5 mmHg, or high: 25 mmHg), the order of which was randomized. Within each ICP level, animals had BP set to either normal or high pressure. Two ICP levels were randomly chosen and run in each animal, and BP was the same for the two ICP conditions. Between the two ICP conditions, a minimum of 30 min was allowed for recovery from the previous IOP elevation. These ICP levels were chosen as we have previously shown that they produce clear differences in the way the rat optic nerve responds to IOP elevation (Zhao et al., 2015). They provide a basis for understanding how changes in blood pressure further impact this relationship.

To assess how ICP and BP influenced the functional impact of IOP elevation, a stepwise IOP protocol was used. IOP was first set to a baseline of 10 mmHg for 10 min before it was gradually increased to 90 mmHg in 10 mmHg increments (each 3 min). Measurements were taken at 2 min after the onset of each IOP step. After completing one series of the IOP step protocol, IOP, ICP, and BP were set back to baseline for 30 min for recovery. It is possible that with the stepwise IOP protocol, the response to each IOP level is impacted by previous levels. Overall, the magnitudes of attenuation that we see for IOP levels from 60 mmHg to 90 mmHg are similar to those previously reported (Bui et al., 2005). In that study, the effect of each IOP level was assessed independently of any other IOP elevation. In addition, the 30 min recovery between ICP levels may not have allowed enough time for the ERG time to recover from the previous stepwise IOP protocol. Based on previous work, the cumulative IOP integral of the stepwise protocol is 1,320 mmHg.min, from which the ERG will have recovered to within 90% of baseline after 30 min (He et al., 2006).

### 2.6 Functional assessment: electroretinography

Retinal function was assessed using a full-field electroretinogram (ERG) as schematized in Figure 1D. As previously reported (Zhao et al., 2019b), scotopic ERGs were recorded following overnight (12 h) dark-adaptation of animals. All preparations were undertaken in a dark room with the aid of a dim red headlight to minimize light exposure during surgery (blood vessel and lateral ventricle cannulation) and ERG electrode placement. Responses were recorded using a set of custom-made chloride silver active and reference (ring-shaped) electrodes placed on the corneal apex and around the sclera at the equator, respectively. A stainless-steel needle electrode (F-E2-30, Grass Telefactor, West Warwick, RI, United States) was inserted subcutaneously into the tail and served as the ground. Light was delivered using calibrated white LEDs (Luxeon LED, Philips® Lumileds Lighting Company, San Jose, CA, United States) embedded into a Ganzfeld sphere (Photometric Solutions International, Huntingdale, VIC, Australia). Stimulation and signal capture were triggered with Scope™ software (ADInstruments). Signals (4 kHz sampling) were acquired with filter settings of 0.3–1,000 Hz (−3 dB) via pre-amplifiers (P511 Amplifier, Grass Telefactor) through an amplifier (ML785 Powerlab 8SP, ADInstruments) and saved for *post hoc* analysis.

At each IOP step, ganglion cell function was assessed by taking the average of 20 flashes (2 s of interstimulus interval) of dim light (−5.25 log cd s/m^2^) to elicit a scotopic threshold response (STR). Immediately after STR acquisition, a moderate flash (−2.48 log cd s/m^2^) was delivered to elicit a rod bipolar cell response (b-wave). At each IOP step, ERG recording took 45 s, allowing at least 135 s for readaptation before the next IOP step/ERG recording (Lee et al., 2023). This interstimulus interval between IOP steps of over 2 min ensured that there was adequate readaptation time. That our STR amplitudes remained stable over IOP levels from 10 mmHg to 50 mmHg would suggest that the interstimulus interval was adequate. Waveforms were analyzed by measuring the amplitude from the baseline to the peak of the positive STR or b-wave (Figure 1E).

Amplitudes were plotted against IOP levels for the two BP groups as well as the three ICP levels. Additionally, during each recording window, the IOP (mmHg), ICP (mmHg), and BP (mmHg) levels were used to derive the following:

$$\text{optic nerve pressure difference }\left(\text{ONPD}\right)=\text{IOP }–\text{ ICP},$$ (1)

$$\text{ocular perfusion pressure }\left(\text{OPP}\right)=\text{BP }–\text{ IOP},$$ (2)

difference between OPP and ICP=BP – OPND (3)

As the episcleral veins are subjected to the tissue pressure in the orbital cavity, we reasoned that a larger difference between the perfusion pressure in the eye and the tissue pressure outside the eye could increase resistance to retinal venous outflow. As such, we added ICP to OPP (Equation 3). For each of the above-derived pressures, values across all animals for a given BP group were placed into 10 mmHg bins (i.e., the 100 mmHg bin would encompass values from 95 mmHg to 104.9 mmHg).

### 2.7 Statistical analysis

Statistical analysis was undertaken using GraphPad Prism (v.10, La Jolla, United States). Only ERG amplitudes from IOP-treated eyes were used in the analysis. Contralateral ERG amplitudes were not used, and the ERG amplitude at baseline IOP in the cannulated eye served as the reference point for statistical comparisons. Data were first checked for normality and homogeneity of variance. Two-way repeated-measures ANOVA was used for group comparisons. Post hoc analysis was conducted using Dunnett’s multiple comparisons (within groups) and Tukey’s multiple comparisons (across groups). Group data are given as mean ± SEM. An alpha value of 0.05 was used to determine statistical significance.

## 3 Results

**FIGURE 2:**
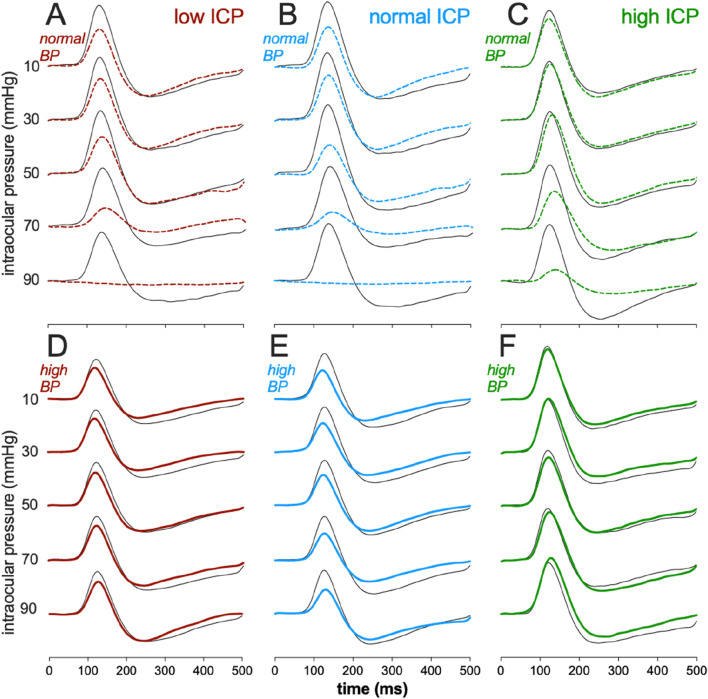
Effect of intracranial pressure (ICP) and blood pressure (BP) on the electroretinogram response to IOP elevation in rat retina. Average scotopic threshold responses (STR) to sequentially high IOP levels at various combinations of ICP and BP. **(A)** Low ICP, normal BP. **(B)** Normal ICP, normal BP. **(C)** High ICP, normal BP. **(D)** Low ICP, high BP. **(E)** Normal ICP, high BP. **(F)** High ICP, high BP. Thick traces: eye undergoing IOP elevation, Thin traces: contralateral control eye.

Figure 2 shows the effect of IOP elevation on STR waveforms. At normal BP levels (Figures 2A–C), higher levels of IOP elevation resulted in greater attenuation of the ganglion cell-mediated STR. However, the degree of STR attenuation was less when ICP was high (25 mmHg). For animals with high BP (Figures 2D–F), basal ERG responses were smaller than those with normal BP. Finally, IOP elevation had very little effect on the ERG in animals with high blood pressure, even at the higher IOP level of 90 mmHg and across all ICP levels.

**FIGURE 3:**
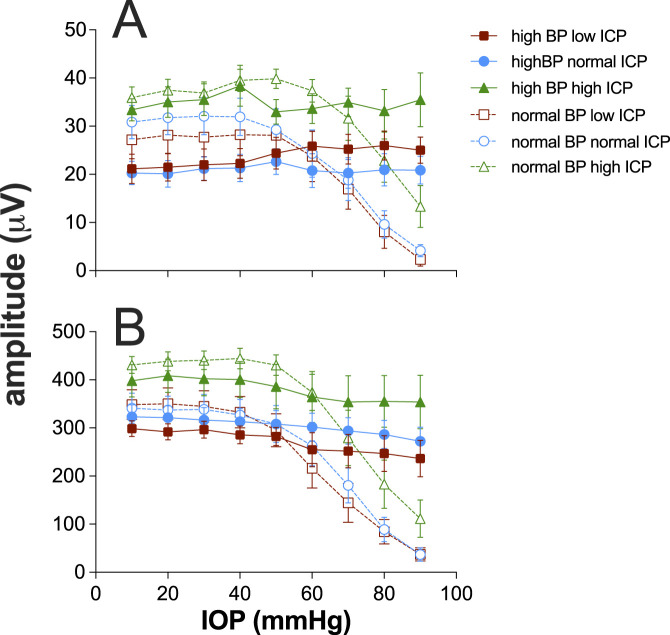
Effect of intracranial pressure (ICP) and blood pressure (BP) on electroretinogram response to IOP elevation in rat retina. **(A)** Group (±SEM) scotopic threshold response (STR) amplitude at various IOP levels at low (squares, n = 7 and 8), normal (circles, n = 8 and 11), and high ICP (triangles, n = 7 and 9). In each case, data are shown for normal (unfilled symbols) and high blood pressure (filled symbols). **(B)** Group (±SEM) bipolar cell b-wave amplitude at various IOP levels at low (squares, n = 7 and 8), normal (circles, n = 8 and 11), and high ICP (triangles, n = 7 and 9). In each case, data are shown for normal (unfilled symbols) and high blood pressure (filled symbols).

Figure 3A summarizes the impact of ICP and BP on ganglion cell function, as measured using the STR full-field ERG. As has been reported (Bui et al., 2005; He et al., 2012) in anesthetized rats with normal BP, IOP elevation (Figure 3A) above 50 mmHg results in attenuation of the STR, and at 90 mmHg, the STR was largely abolished in the low ICP group, and least affected in the high ICP group. Two-way ANOVA confirms that there was a significant difference between ICP levels (F_2,24_ = 4.3, p = 0.024) without significant interaction between ICP and IOP (F_2,8_ = 0.64, p = 0.85).

When BP was elevated, there was a significant reduction in STR amplitude at baseline IOP of 10 mmHg (F_1,44_ = 6.4, p = 0.015). This effect appeared to be smaller in magnitude for the high ICP group (Figure 3A); however, there was not a significant interaction between blood pressure and ICP level (F_2,44_ = 0.9, p = 0.4). These data would suggest that very high perfusion pressures are not optimal for ganglion cell function. Across the three different ICP levels, the STR was relatively well preserved with increasing IOP elevation, with only a small attenuation of the STR above IOP levels of 70 mmHg, which was not statistically significant (F_1,44_ = 1.8, p = 0.2).

Figure 3B shows data for the bipolar cell-mediated ERG b-wave. The trends evident for the STR are similar to those shown here for the b-wave, with the b-wave being significantly larger for the high ICP group than the others (F_2,24_ = 4.3, p = 0.01). At high blood pressure, there was no difference between ICP groups (F_2,24_ = 4.3, p = 0.07). Unlike the STR, which showed lower baseline amplitudes with higher blood pressure, there was no difference between baseline b-wave amplitudes for normal and high blood pressure groups across the three ICP levels (F_1,44_ = 2.2, p = 0.14).

**FIGURE 4:**
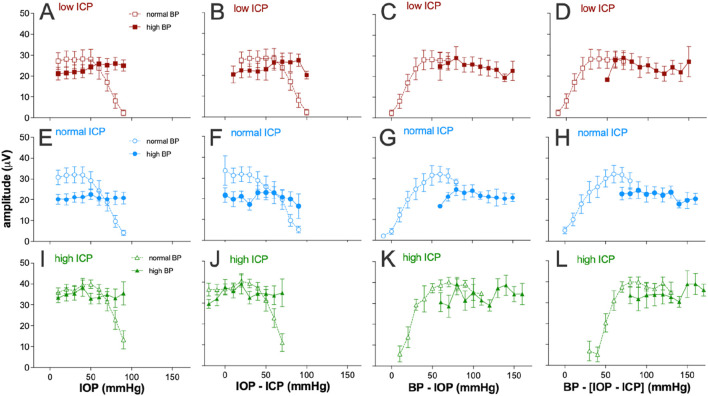
Electroretinogram response to IOP elevation in rat retinas plotted against various eye pressure metrics. Averaged group (±SEM) ganglion cell-mediatedscotopic threshold response (STR) amplitude at various IOP levels at low (squares, n = 7 and 8), normal (circles, n = 8 and 11), and high ICP (triangles, n = 7 and 9). In each case, data are shown for normal (unfilled symbols) and high blood pressure (filled symbols). Each column plots the group dataagainst a different metric of tissue pressure: **(A,E,I)** IOP, **(B,F,J)** optic nerve pressure difference (IOP–ICP), **(C,G,K)** ocular perfusion pressure (BP–IOP), and **(D,H,L)** ocular perfusion pressure combined with ICP [BP – (IOP–ICP)].

Figure 4 considers various expressions of pressure and how they might account for the pattern of ganglion cell responses observed as the three pressures (IOP, ICP, and BP) were systematically manipulated. To facilitate visualization, data for normal and high blood pressure groups are shown as unfilled and filled symbols, respectively, on the same axis. Plotting ganglion cell amplitude against IOP alone (Figures 4A,E,I) cannot account for the disconnect in ganglion cell amplitude patterns seen when comparing the normal and high blood pressure groups. This was also the case when data were plotted against the optic nerve pressure difference (Figures 4B,F,J; Equation 1).

Ocular perfusion pressure (OPP, Equation 2) is a useful surrogate for the local difference between the pressure in the vasculature (BP) and surrounding ocular tissues (IOP). This has been used as a predictor of nutrient supply to support retinal function. Not surprisingly, a more continuous function emerges when ganglion cell amplitudes for both normal and high BP groups are plotted against OPP for all three ICP levels (Figures 4C,G,K). However, when ICP was normal, with IOP elevation, the high BP group (Figure 4G, filled symbols) showed more STR attenuation that would be predicted based on the STR returned for the same OPPs from the normal BP groups (unfilled symbols). This was apparent at the lowest perfusion pressures in this group, between 60 mmHg and 70 mmHg. This might suggest that at the highest IOP levels (70–90 mmHg), IOP-mediated injury both includes a vascular component that can be overcome by high BP and a mechanical compression component, which cannot be overcome by higher blood pressure.

Another potential determinant of nutrient supply to the retina may be the difference between the perfusion pressure in the eye and ICP (Equation 3). That is, a lower ICP would result in a larger difference between perfusion inside and outside the eye. To address this possibility, STR amplitudes for the various groups were plotted against the difference between OPP and ICP, with a high ICP producing a smaller pressure drop between intraocular and extraocular compartments. Considering ICP in this way does not appear to produce better alignment between the high and normal blood pressure groups (compare Figures 4D,H,L).

## 4 Discussion

This study considered the acute effects of modifying BP and ICP on the way that retinal function responds to IOP elevation. As has been reported previously in rats (Bui et al., 2005; He et al., 2012; Zhao et al., 2015), at normal BP, higher levels of IOP elevation result in greater attenuation of retinal function, both in terms of the bipolar cell b-wave and the ganglion cell STR. Moreover, we confirm that acute high BP reduces the functional attenuation seen in the ERG by IOP elevation (He et al., 2012). The rate of functional decline can be explained by reduced blood flow (Karasinska et al., 2013; Xu et al., 2019; Zhao et al., 2020; Zhi et al., 2012) along with biomechanical effects of very high IOP levels (He et al., 2013).

We extend these observations to show that compared with normal blood pressure, the higher blood pressures used in this study are not optimal for ganglion cell function. Indeed, with IOP at baseline, our analysis showed that having high blood pressure resulted in significantly smaller ganglion cell STR responses. Importantly, this reduction in amplitude was not seen for the rat ERG b-wave. A decline in neural function with high blood pressure and high blood flow velocity is consistent with previous modeling in the brain (Buxton and Frank, 1997; Hayashi et al., 2003) and retina (Bek and Jeppesen, 2021), showing that oxygen extraction declines at high blood pressure. It is of interest that this phenomenon is not evident for the b-wave. It is likely that the b-wave generated by bipolar cells might benefit from increased oxygen diffusion arising from high flow through the greater vascular volume of the choroid (Reiner et al., 2010). Indeed, Yu et al., 1994 showed that oxygen diffusion from the choroid resulted in the relationship between oxygen tension and systemic BP in rats being linear for the deep capillary layer of the retina and in the choriocapillaris. In contrast, the inner retinal vasculature supporting the ganglion cell layer shows a saturating profile with only a small increase in oxygen tension between systemic blood pressures of 100 mmHg and 150 mmHg.

Interestingly, we also found that acutely elevated ICP resulted in larger ganglion cell STR amplitudes but similar b-wave responses (Figure 3). This differs from data reported previously by Zhao et al. (2015), which found that elevated ICP resulted in larger ganglion cell STR and b-wave amplitudes, with the ganglion cell response being more sensitive. However, those data were acquired from younger animals (3 months), which raises the possibility that any acute benefit to inner retinal function provided by higher ICP may be more important in the older animals (18 months) used in this study. Why high ICP would selectively benefit ganglion cell function is unclear. Whether higher ICP in these older eyes better supports blood flow and oxygen extraction remains to be investigated. Additionally, as older eyes are more susceptible to deformation at the lamina cribrosa (Albon et al., 2000), higher ICP may be protective of ganglion cell function in older animals by preserving the architecture of the connective tissues supporting RGC axons.

**FIGURE 5:**
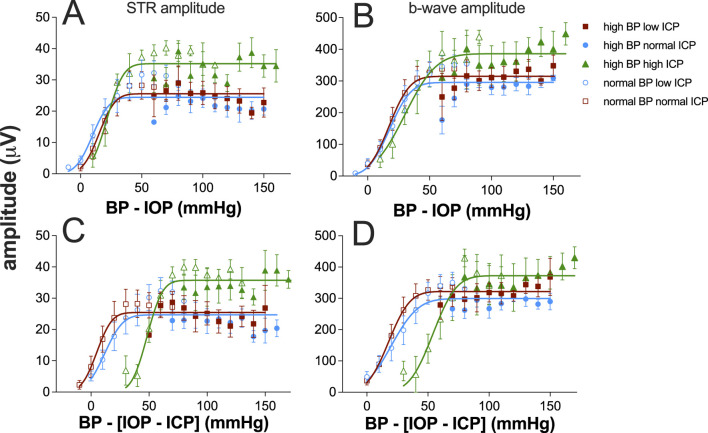
Ganglion cell and bipolar cell responses plotted against ocular perfusion pressure (OPP, **(A, B)** or against the difference between OPP and ICP **(C, D)**. Group (±SEM) scotopic threshold response (STR) amplitude at various IOP levels at low (squares, n = 7 and 8), normal (circles, n = 8 and 11), and high ICP (triangles, n = 7 and 9). In each case, data are shown for normal (unfilled symbols) and high blood pressure (filled symbols). **(A, C)** STR data are replotted from Figure 4. **(B, D)** Bipolar cell b-wave amplitude. Data for each ICP level (both normal and high BP) were fit using a cumulative Gaussian function.

A key aim of the study was to consider how blood pressure and ICP interact to support retinal function. Figure 5 summarizes all data from all groups for both STR and the b-wave amplitudes against either ocular perfusion pressure (OPP) or against the OPP and ICP difference. These two pressure expressions were described using a cumulative Gaussian function, and the goodness-of-fit was compared using an F-ratio. There was no statistically significant difference between the two pressure expressions (Supplementary Table S1). Thus, adding ICP to the pressure relationship did not account for more of the variability in the data. This would argue that the major mechanism at play is vascular autoregulation (He et al., 2013). In both cases, neither expression can account for the increase in STR induced by high ICP. These data suggest that for rat ganglion cell function, ICP acts in a complex way and is not simply a modifier of either IOP or BP. For example, the Cushing reflex would result in decreased blood flow with high ICP (Dickinson, 1990); however, one would not expect an increase in function with decreased blood flow. The data also suggest that high ICP acutely impacts the STR and thus the inner retina where this response is generated (Bui and Fortune, 2004), more than the b-wave, which is dependent on both outer and inner retinal blood supply (Bui et al., 2005).

It is important to note that the data reported here are for older rats. It is known that beyond 12 months of age, the way in which ICP modification affects retinal function differs from younger animals (Zhao et al., 2018). As such, it will be important to directly compare younger and older animals. Indeed, as mentioned above, ICP modification in younger rats did not appear to systematically increase the ganglion cell-driven STR (Zhao et al., 2015), as was seen here. To relate pressure modification to function, it will be important to consider the measurement of optic nerve deformation, blood flow (both in the choroid and retina), and retinal oxygen profiles in parallel cohorts. This study has been limited to two blood pressure levels and three ICP levels. Data across a wider range of pressure levels will provide significant inputs into more sophisticated models than the simple linear expressions of pressure used here. Finally, modifying BP can impact ICP, and vice versa; thus, studies should employ approaches that allow more precise control of these pressures, perhaps via servo null feedback, to more fully model and understand how they modulate the effect of IOP elevation.

## 5 Conclusion

We show that BP and ICP both modify the impact of IOP elevation on retinal function. This impact was greater for ganglion cells than bipolar cell-mediated ERG responses. Acute increases in ICP levels appear to increase the ganglion cell STR response. Overall, OPP provides a useful expression to account for changes in retinal function across a range of IOP, BP, and ICP levels in older rats. The inclusion of ICP in a pressure equation does not appear to better explain retinal function.

## Data Availability

The original contributions presented in the study are included in the article/Supplementary Material; further inquiries can be directed to the corresponding author.
