# Supplementary material for: Rat retinal function attenuation with IOP elevation is impacted by both blood pressure and intracranial pressure
Source: Front Physiol. 2025 Jun 18;16:1566032. doi: 10.3389/fphys.2025.1566032 (PMC12213785; doi:10.3389/fphys.2025.1566032)
Supplement: Supplementary file 1 [file DataSheet1.docx]

***Supplementary Figure 1:*** *Averaged (± SEM) mean arterial blood pressure at baseline and at each IOP level. Data are shown for group with low (squares, n =7 and 8), normal (circles, n = 8 and 11) and high ICP (triangles, n = 7 and 9). In each case data are shown for normal (unfilled symbols) and high blood pressure (filled symbols).*


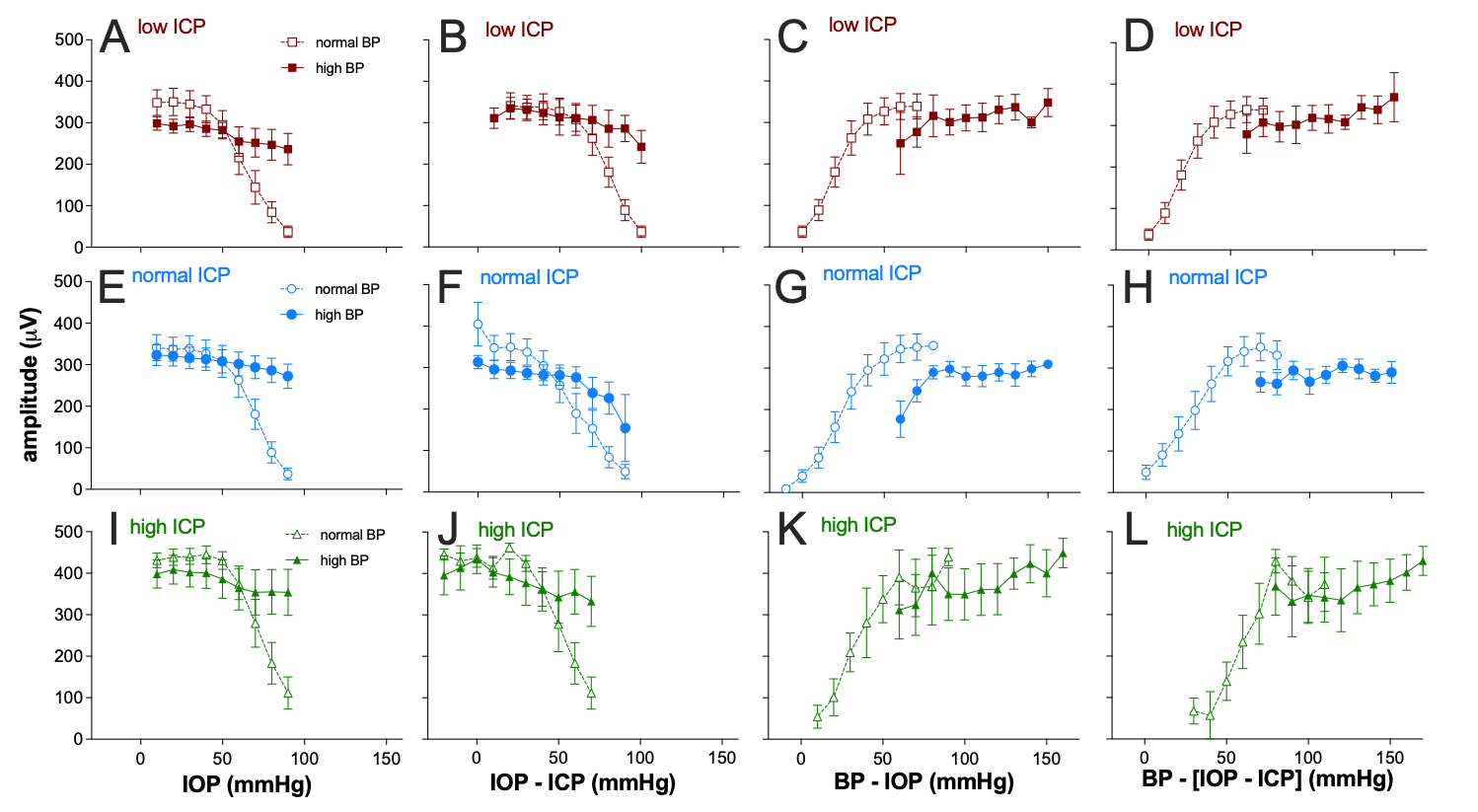


***Supplementary Figure 2:*** *b-wave electroretinogram response to IOP elevation in rat retina plotted against various eye pressure metrics. Averaged group (± SEM) bipolar cell mediated b-wave amplitude at various IOP levels at low (squares, n =7 and 8), normal (circles, n = 8 and 11) and high ICP (triangles, n = 7 and 9). In each case data are shown for normal (unfilled symbols) and high blood pressure (filled symbols). Each column plots the group data against a different metric of tissue pressure i) IOP, ii) optic nerve pressure difference (IOP – ICP), iii) ocular perfusion pressure (BP – IOP) and iv) ocular perfusion pressure combined with ICP (BP – [ICP – IOP]).*

***Supplementary Table 1:*** *Cumulative normal gaussian fit to combined scotopic threshold response (STR) and b-wave amplitude group data across both normal and high blood pressure groups for low, normal and high ICP. Fits can be visualised in Figure 5. Model 1: amplitudes were plotted against ocular perfusion data (BP-IOP). Model 1: amplitudes were plotted against ocular perfusion data minus ICP (BP – [IOP-ICP]). Models were compared using a F-ratio. There was no significant different in terms of goodness-of-fit between models 1 and 2.*

| **STR amplitude** | | | | | | |
| --- | --- | --- | --- | --- | --- | --- |
|  | **Model 1: BP-IOP (mmHg)** | | | **Model 2: BP-[IOP-ICP] (mmHg)** | | |
| Gaussian Best-fit values | Normal ICP | Low ICP | High ICP | Normal ICP | Low ICP | High ICP |
| Mean | 9.80 | 9.97 | 21.69 | 11.82 | 5.133 | 48.34 |
| Standard deviation | 10.8 | 10.3 | 10.5 | 12.3 | 10.01 | 10.4 |
| Amplitude | 24.4 | 24.4 | 35.1 | 24.6 | 25.4 | 35.7 |
| Goodness of Fit |  |  |  |  |  |  |
| Degrees of Freedom (df) | 17 | 16 | 18 | 16 | 16 | 19 |
| R-squared | 0.73 | 0.60 | 0.83 | 0.65 | 0.69 | 0.89 |
| Sum of Squares (SS) | 330 | 328.2 | 240.5 | 278.4 | 145.7 | 189.9 |
| Mean Square (SS/df) | 19.4 | 20.5 | 13.3 | 17.4 | 9.1 | 9.99 |
| F-ratio (MS1/MS2) | 0.89 | 0.44 | 0.74 |  |  |  |
| p-value (Model1 vs Model2) | 0.86 | 0.48 | 0.93 |  |  |  |
| **B-wave amplitude** | | | | | | |
|  | **Model 1: BP-IOP (mmHg)** | | | **Model 2: BP-[IOP-ICP] (mmHg)** | | |
| Gaussian Best-fit values | Normal ICP | Low ICP | High ICP | Normal ICP | Low ICP | High ICP |
| Mean | 17.67 | 17.26 | 29.96 | 20.08 | 17.77 | 54.03 |
| Standard deviation | 13.52 | 13.17 | 20.04 | 17.23 | 13.71 | 15.08 |
| Amplitude | 295.4 | 315 | 386.1 | 299.2 | 322.1 | 372.5 |
| Goodness of Fit |  |  |  |  |  |  |
| Degrees of Freedom | 17 | 15 | 17 | 15 | 15 | 16 |
| R-squared | 0.85 | 0.92 | 0.90 | 0.90 | 0.94 | 0.92 |
| Sum of Squares | 27870 | 9357 | 20244 | 11218 | 6820 | 17812 |
| Mean Square | 1639.4 | 623.8 | 1190.8 | 747.8 | 454.6 | 1113.2 |
| F-ratio (Model1/model2) | 0.45 | 0.72 | 0.93 |  |  |  |
| p-value (Model1 vs Model2) | 0.50 | 0.86 | 0.84 |  |  |  |
